# Epithelial-Dermal Immune Memory: Tracking *Staphylococcus aureus*-Induced Trained Immunity in the Progression of Chronic Skin Inflammation

**DOI:** 10.3390/ijms27156760

**Published:** 2026-07-28

**Authors:** Vidhya Prakash, Shivani Chalil Velluva, Shiv Shankar Sreekumar, Nidhi Jayaprakash, Nandana S. Pillai, Noura Nizar, Nikita Arun, Kalyani Arun, Mohammed Aslam, Nidhisha Babysulatha Sasidharan, Parvathy Venugopal, Aparna Shankar, Bipin G. Nair

**Affiliations:** 1 School of Biotechnology, Amrita Vishwa Vidyapeetham, Clappana P.O, Kollam 690525, Kerala, Indiaparvathyv@am.amrita.edu (P.V.); bipin@am.amrita.edu (B.G.N.); 2 Centre of Excellence in Microbiome, Government of Kerala, Trivandrum 695585, Kerala, India

**Keywords:** trained immunity, atopic dermatitis, cellulitis, chromatin accessibility, epigenetic reprogramming, short-chain fatty acids

## Abstract

The clinical paradigm distinguishing chronic atopic dermatitis (AD) from acute bacterial infections is established by the emerging evidence which focuses on persistent innate immune memory as a central pathobiological factor mediating inflammatory skin diseases. While AD is classically defined by genetic susceptibility and epidermal barrier failure, progression of deep dermal *Staphylococcus aureus* invasion, which is characteristic of bacterial infections like cellulitis, implies a significant acute infectious condition resulting in significant tissue damage, which drives extensive innate immune reprogramming. Recent studies have established a bidirectional relationship between these conditions, yet the underlying molecular mechanisms remain undefined. However, trained immunity, governed by epigenetic and metabolic reprogramming, has now emerged as a critical mediator of this interaction. This review attempts to explore the molecular foundations of trained immunity as a mechanistic link between acute infectious conditions and chronic skin inflammation. The critical involvement of bacterial virulence factors and pattern recognition receptor signalling pathways, which instigate persistent innate memory, predisposes the tissue to recurrent infections and aggravated inflammatory responses. Furthermore, the persistent involvement of innate immune reprogramming in facilitating a mechanistic continuum between cellulitis and chronic dysregulation, characteristic of atopic dermatitis, is discussed. In continuum, we propose emphasising emerging therapeutic strategies targeting epigenetic checkpoints and metabolic rewiring to develop novel immunomodulatory interventions to combat infectious and inflammatory skin disorders.

## 1. Introduction

Skin is an active immunological organ that incorporates environmental information to preserve the barrier and maintain the immune system at homeostasis through the coordinated actions of keratinocytes, macrophages, dendritic cells, and innate lymphoid cells [1,2,3]. Contrary to the traditional concept that immunological memory is restricted to adaptive immunity, emerging evidence indicates that many innate immune cells are capable of undergoing long-lasting epigenetic and metabolic reprogramming, termed “trained immunity”, resulting in long-term functional changes irrespective of whether the triggering stimulus is resolved [4,5,6,7]. A network of memory in relation to tissue function is established through keratinocyte transcriptional imprinting, converging neuroimmune pathways, and the microbiome-derived signals.

Skin diseases have conventionally been classified into infectious and inflammatory categories depending on the underlying mechanism. The innate immune responses in the skin are initiated in the epidermis through pattern-recognition receptor (PRR) activation on aggravated keratinocytes and group 2 innate lymphoid cells (ILC2s). While apparent epidermal pathogen colonisation exerts a localised training stimuli, the breakdown of resolving mechanisms facilitates deep paracellular pathogen invasion. This transition, inferred as deep-tissue cellulitis, further recruits a greater sequence of immune cells including dermal macrophages and monocytes. This further supports the extensive, enduring systemic evidence of innate immune memory [8]. This complex interplay creates an association between recurrent bacterial infection, i.e., cellulitis, and a prolonged inflammatory state, increasing the risk for developing chronic inflammatory diseases like atopic dermatitis (AD) [9,10]. Cellulitis is a bacterial infection of the deeper dermis, mostly caused by *Staphylococcus aureus* and *Streptococcus pyogenes*, often characterised by erythema, warmth, and swelling [11]. Atopic dermatitis, on the other hand, is a chronic, inflammatory disease that impacts the integrity of the epidermal barrier, a consequence of an underlying genetic predisposition or immune defect that is often associated with Th2 polarisation and IgE-mediated response [12]. However, this classification into a dichotomy has been under revision and challenged, given the growing evidence that microbial exposure can induce durable alterations to immunity beyond resolution of initial infection [13]. Persistent colonisation with *Staphylococcus aureus* in the epidermis, clinically manifesting as impetigo, causes deterioration of epidermal integrity and exacerbates Th2-skewed inflammation via a toxin-driven activation of the immune system, culminating in the chronic inflammatory responses of AD [14].

Recent evidence suggests ‘trained immunity’ enhances responsiveness to secondary stimuli, and while protective in host defence, this may predispose tissues to chronic inflammation [15]. These observations shed light on the proposal that recurrent bacterial infections, including cellulitis, may provide a constant source of training stimulus for the immune and epithelial cells of the skin. This impacts deep-rooted alterations to epigenetics and metabolism, lowering the threshold required to activate the immune system, thereby increasing the response to inflammation. Therefore, chronic exposure to pathogens may establish an ongoing immune memory between the acute infection and the development of chronic inflammatory disease, such as atopic dermatitis.

Overall, this review attempts to synthesise a comprehensive evaluation of the molecular foundations of trained immunity with specific reference to epigenetic and metabolic reprogramming, recalling the dynamism and expansive immunological memory of the skin. By comparing the molecular triggers leading to cellulitis and the subsequent imprinting of innate immune cells, a continuum model of how exposure to recurrent skin infections as a primary training event provides a preface to the chronic dysregulated inflammatory responses in atopic dermatitis is discussed. Furthermore, the focus has also been shifted to resolve the failure of traditional antibiotic therapies in downregulating persistent inflammation by emphasising the critical role of epigenetic modulators and postbiotic interventions in manipulating maladaptive conditions, thereby maintaining cutaneous homeostasis.

## 2. Hallmarks of Trained Immunity

**Figure 1:**
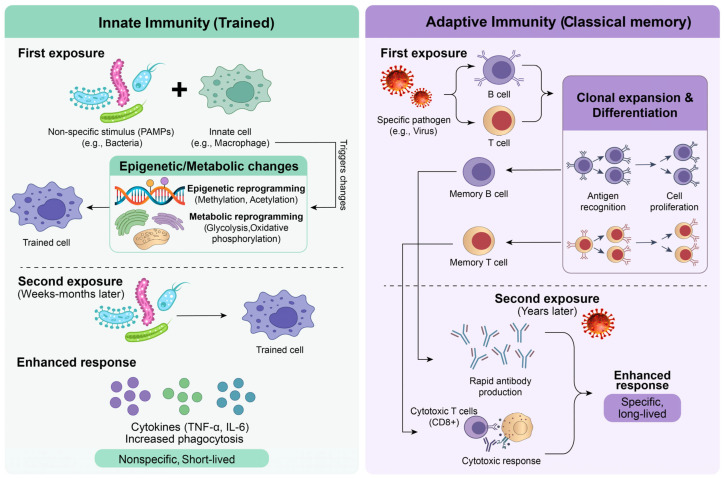
Difference between adaptive immune memory and trained innate immune response: In innate immunity, first exposure to non-specific stimuli (PAMPs) activates cells (e.g., macrophages), inducing epigenetic (methylation and acetylation) and metabolic reprogramming that generates a trained, memory-like phenotype. On second exposure (weeks to months), these cells mount a faster, amplified, broad, but short-lived response with increased cytokine production (TNF-α and IL-6) and enhanced phagocytosis. In adaptive immunity, first exposure involves antigen-specific recognition by B and T lymphocytes, leading to clonal expansion and formation of effector and memory cells. Upon re-exposure (months–years), memory cells trigger rapid, highly specific, and long-lasting responses, including antibody production by B cells and cytotoxic activity of CD8^+^ T cells.

Within the evolving paradigm of dermatological research, which holds foundational history in classic immunology, boundaries have been set between infectious cellulitis and chronic inflammatory atopic dermatitis. However, a significant paradigm shift has focused attention on the critical role of innate immunity in atopic dermatitis. There is a deviation in the immunological imprint from the fundamental dogma pointing to the concept of trained immunity, depicting that the innate immunity inherits a form of long-term inflammatory memory. Immune memory has traditionally been a feature of the adaptive immune system and is mediated by B and T lymphocytes that exhibit antigen-specificity. Following exposure to an antigen, these lymphocytes can proliferate and differentiate into long-lived memory cells, which enable the immune system to respond more efficiently to antigens that are encountered again. The antigen-specificity and longevity of these immune cells distinguish the adaptive immune system from the innate immune system [16]. The core differences describing the concept of adaptive and trained innate immune responses are represented in Figure 1. Historically, this highly enduring and antigen-specific memory was considered an exclusive hallmark of the adaptive immune system. However, recent studies have altered this perspective by introducing the concept of imprinting of trained immunity in skin cells. Acute cellulitis imprints a long-term innate memory post the primary bacterial exposure, and the pathogens associated enhance chromatin remodelling, increasing skin sensitivity and facilitating the entry of secondary pathogens, promoting chronic inflammatory conditions like atopic dermatitis.

### 2.1. Epigenetic Mechanisms Underlying Training

**Figure 2:**
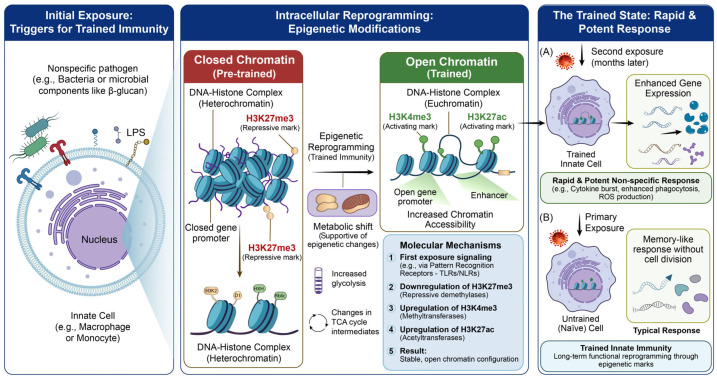
Epigenetic modification driving chromatin accessibility in trained immunity: The initial exposure of innate immune cells (e.g., monocytes/macrophages) to non-specific stimuli such as microbial components (e.g., LPS and β-glucan) triggers intracellular signalling via pattern recognition receptors. This induces epigenetic and metabolic reprogramming, shifting chromatin from a closed (heterochromatin; marked by repressive H3K27me3) to an open state (euchromatin; enriched with activating marks like H3K4me3 and H3K27ac), thereby increasing gene accessibility. These changes support metabolic shifts such as enhanced glycolysis and TCA cycle intermediates. Upon secondary exposure, trained innate cells exhibit a rapid and amplified response with increased cytokine production, phagocytosis, and ROS generation, unlike naive cells, which show a typical primary response representing trained immunity.

The adaptive immunity mainly focuses on clonal expansion and somatic gene arrangement to attain antigen specificity, whereas the trained immunity focuses on robust functional reprogramming. This process is initiated when innate immune cells, such as monocytes and macrophages, encounter microbial stimuli that do not specifically target any cellular components (e.g., bacterial components like LPS or β-glucan), which act as the primary triggers for trained immunity. This is governed by epigenetic molecular drivers, specifically histone markers like H3K4me3 and H3K27ac [17]. This enhances chromatin accessibility for maintaining long-term inflammatory signalling [4,18]. This allows the chromatin to maintain a primed, accessible state even after the first pathogen entry is cleared, allowing trained innate immune cells to mount enhanced responses to subsequent stimulation through sustained epigenetic remodelling and chromatin accessibility [1]. This enables the chromatin to retain a prime accessible state even after clearance of initial pathogen entry (such as *Staphylococcus aureus* or *Cutibacterium acnes*). Another mode of transition that is supported is through the reprogramming of the cell’s metabolism to promote processes like glycolysis and the TCA cycle, as the intermediates created during these processes act as cofactors for the enzymes which modify histones to stabilise the open chromatin structure. This trained immune response results in an increased transcriptional activation of pro-inflammatory cytokines in response to heterologous stimuli, such as secondary microbial challenges. Moreover, novel data suggest that systemic signals induced by the gut microbiota may modulate immune responses at distant barrier sites like the skin through the gut–skin axis, thus potentially contributing to trained immune responses in cutaneous inflammatory disorders [19]. Upon secondary exposure, innate immune cells that have been trained exhibit enhanced gene expression, cytokine secretion, and antiviral functions compared to naïve cells, which only exhibit a typical primary immune response. Epigenetic modifications mediating chromatin accessibility in mediating trained immunity are represented in Figure 2.

The fundamental mechanism by which trained skin maintains cutaneous hypersensitisation is accounted for by the enhanced chromatin accessibility, which persists in primed as well as open chromatin configurations. This justifies the fact that chronic inflammation in AD-like phenotypes is attributed to the continuous expression of pro-inflammatory cytokine loci, which in turn demarcates transcriptional activity and persistent inflammatory signalling [20]. For instance, experimental evidence also proved that the incorporation of specific postbiotics and phytochemicals could act as epigenetic modulators promoting chromatin restoration. By targeted inhibition of NLRP3/caspase-1/IL-1β signalling, these agents mediate the DNA accessibility persistently, which otherwise drives chronic inflammatory conditions [21].

### 2.2. Metabolic Rewiring of Trained Cells

Furthermore, these underlying mechanisms tend to facilitate molecular rewiring of metabolic pathways, resulting in a shift in cellular metabolism that promotes glycolysis, glutaminolysis, and the mevalonate pathway. Most homeostatic cells depend on oxidative phosphorylation, causing increased glycolytic flux, necessitating effective cellular priming to maintain the cell in a persistent primed state [22]. This metabolic overdrive may trigger an increased production of proinflammatory cytokines, IL-1β, when exposed to a secondary pathogenic challenge which delineates chronic cutaneous inflammation. In this context, even though glycolysis serves as a primary energy source, essential metabolic intermediates are from the mevalonate pathway [23]. This pathway produces metabolites that could function as critical cofactors for epigenetic enzymes like histone acetyltransferases, which further facilitate key histone modifications. For instance, H3K4me3 and H3K27ac promote chromatin decondensation, thereby increasing accessibility at pro-inflammatory loci [24]. In line with this mechanism, trained immunity has been associated with sustained enrichment of activating histone marks such as H3K4me3 and H3K27ac at promoters and enhancers of inflammatory genes, resulting in enhanced transcriptional responses upon secondary stimulation [25].

Pathobionts are commensals which, in response to changes in the local microenvironment, turn into opportunistic pathogens and are often the cause of chronic dermatoses. Prolonged microbial exposure can contribute to a sustained inflammatory state through trained immunity in skin-resident innate immune cells. Under conditions of cutaneous dysbiosis and barrier dysfunction, pathobionts and their pathogen-associated molecular patterns (PAMPs) activate pattern recognition receptors (PRRs), further enhancing inflammatory responses [26]. Moreover, the impact of antibiotics on intestinal microbiota may lead to long-lasting dysbiosis and systemic immune disturbances that could also influence skin homeostasis through microbiome-mediated immune regulation along the gut–skin axis [27,28]. Additional evidence suggests that gut microbiome dysregulation contributes to the development and persistence of inflammatory skin diseases by modulating immune responses at distant cutaneous sites [29].

New therapeutic strategies are being developed around modulating maladaptive trained immunity to prevent exaggerated inflammatory responses in the skin. Targeting the epigenetic and metabolic mechanisms that uphold inflammatory memory may reinstate cutaneous immune homeostasis, reduce chronic inflammation, and improve tissue repair. Thus, reverting chronic inflammatory programming is an appealing strategy to manage chronic skin infections and the systemic immune dysregulation that accompanies them [30,31].

## 3. Skin as an Immunological Memory Organ

Defining Cellular Components in Skin Immune Memory.

Inflammation is the primary factor triggering relapsing dermatological conditions. The skin, by virtue of being the largest and external organ, serves as a physical barrier against terrestrial invasions. Apart from being the greatest immunological footprint, the skin is the crucial site of inflammatory and autoimmune diseases such as psoriasis, atopic dermatitis, cutaneous lupus, and alopecia areata [32].

### 3.1. Keratinocytes as Immune Sentinels

Apart from their structural function, keratinocytes residing in the epidermis are considered to be the primary immune sentinels. Upon barrier breach by pathogens, keratinocytes utilise Toll-like receptors (TLRs) to initiate the NF-κB pathway, which in turn triggers pro inflammatory cytokine activation, specifically IL-1β and TNF-α, along with beta-defensins [33]. Further modulation is activated by the p38MAPK pathway by promoting chemokine secretion (CCL20), facilitating dendritic cell recruitment. However, in chronic conditions, these cells follow a diversified pattern, where they exhibit immunological plasticity by upregulating MHC class II molecules, which in turn present antigens to CD4 cells [34]. This pattern is mediated by the JAK–STAT axis, wherein STAT3 modulates barrier repair, whereas STAT1 mediates antiviral defence. More evidently, recurring stimulation of the cells induces epigenetic imprints specifically through H3K27 acetylation in specific gene loci, promoting a chromatin state identical to trained immunity. Furthermore, these cells actively maintain the local T cell reservoir by secreting homeostatic cytokines like IL-7, IL-15 and release alarmins (TSL, IL-33 and IL-25) which activate innate cells, specifically ILC2s [35].

### 3.2. Langerhans Cells (LCs) and Dermal Macrophages

**Figure 3:**
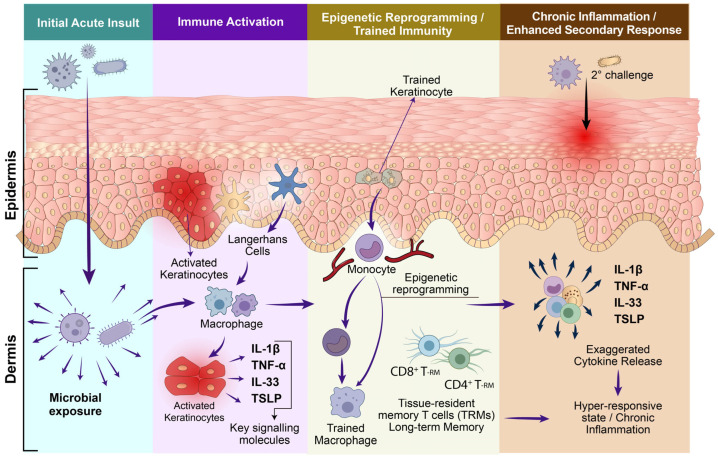
Cellular components and mechanisms in skin immune memory: Initial immune activation is triggered when microbes invade skin, and the irritated keratinocytes residing in the epidermis and resident immune cells (macrophages and Langerhans cells) in the dermis are recognised by the pattern recognition receptors (PRRs). This is followed by cytokine release by the resident immune cells such as IL-1β, TNF-α, IL-33, and TSLP that initiate the immune response to the invading microbes. This in turn activates memory, where the innate immune system cells undergo metabolic and epigenetic alterations following microbe recognition with the additional establishment of CD4+ and CD8+ tissue-resident memory T cells in the skin. Finally, following the initial activation of the immune system and the establishment of memory cells in the skin, the immune system rapidly initiates the cytokine response during secondary exposure, resulting in chronic inflammation.

LCs and dermal macrophages comprise the primary phagocytic network of the skin. LCs are often characterised by CD207, which is a langerin marker and C-type lectin receptor. They maintain tissue homeostasis in a steady state but adapt to transition into a pro-inflammatory phenotype (IL-12) when activated. LCs, derived from the embryonic yolk sac precursors, are sustained by local self-renewal rather than systemic recruitment [36]. Similar characteristics are demonstrated by dermal macrophages, which are known for their high capacity for long-term self-renewal, showing resilience to inflammatory challenges [37]. They are evident players in tissue imprinting, which creates a phenotypic diversity and lineage-specific enhancer blueprint. Emerging evidence shows that these macrophages can retain their metabolic and epigenetic properties from previous inflammatory memory, often consistent with trained immunity traits. Moreover, mast cells and innate lymphoid cells build overlapping connections that stabilise local immunity. Histamines, proteases, and inflammatory mediators are released by mast cells upon activation and engage in crosstalk with group 2 innate lymphoid cells via IL-33 and other alarmins [38]. Skin ILC2 populations show tissue-specific heterogeneity shaped by local signals [39]. More importantly, epidermal ILC2 populations and keratinocytes, which are hyper-responsive, are considered to be the primary immune sentinels recognising initial surface-pattern recognition. However, in deep barrier-invasive wounds, the inflammatory cascade remains within the dermis to demonstrate longer memory persistence over weeks to months. Thus, dermal macrophages and traversing monocyte-derived populations express better capacity for localised self-renewal and structural tissue imprinting. Following translocation of pathogens like *S. aureus* in deep tissues, these cells undergo extensive epigenetic and metabolic reprogramming, thereby reducing the subsequent threshold for non-specific inflammatory reactivity [40,41]. In atopic dermatitis and related disorders, epigenetic accessibility of cytokine loci such as IL-4 and IL-13 promotes rapid cytokine release [42]. Together, mast cell–ILC circuits establish type 2-biased inflammatory microenvironments within the skin [43,44]. An illustration representing the cellular components in skin immune memory and their role in chronic inflammation is represented in Figure 3.

### 3.3. Mechanisms of Immune Memory in Skin

The longevity and morphology of these resident cells foster the autonomous function of the skin, without the expense of a continuous replenishment systemically. It is well established that dermal and epidermal macrophages undergo self-renewal in specific tissue niches. Following an inflammatory response, epigenetic imprinting promotes proinflammatory responses, reinforcing the concept of trained immunity. However, the tissue-based specification of epidermal TRM cells is confined to localised signals, predominantly, TGF-β and PD-1 pathways [45]. These cells, by virtue of their well-regulated microenvironments, promote metabolic adaptation for long-term survival. Further trophic support is provided by keratinocyte-derived cytokines, enhancing the survival of resident cell populations. Furthermore, in contact hypersensitivity models, CD4 TRM cells express their ability to colocalise with Langerhans cells, functioning independently of the circulating myeloid recruitment [46]. Thus, TRM cells serve as immediate response indicators in barrier tissues, making them potential protective agents as well as activators of chronic skin pathology. Moreover, PD-1 and TGF-β are fundamental players in epidermal localisation of these cells. It is also noteworthy that diversity of CD8-TRM cells is also imprinted spatiotemporally, with progenitors and distinct differentiated states occupying specific barrier tissues [47]. These observations thus redefine the skin as a self-sustained immunological organ rather than a mere physical barrier.

## 4. Bacterial Triggers of Trained Immunity in Cellulitis

### 4.1. Clinical Landscape and Recurrence of Cellulitis

The orchestration of innate immune memory mediated by the skin resident cells is now well established, which necessitates the characterisation of mechanisms by which bacterial cellulitis initiates and sustains the trained state. Cellulitis is a frequently occurring bacterial infection that targets the skin and the subcutaneous layers, primarily by *Streptococcus pyogenes* and *Staphylococcus aureus* with clinical symptoms including localised swelling, pain, redness, and warmth. If left untreated, abscess formation, sepsis, or infections that penetrate deeper into the tissues are manifested [48]. Clinically, susceptibility to these types of invasive deep-tissue infections tends to develop with an underlying altered cutaneous microenvironment after primary barrier disruption. There is a deep pathobiological analogy between different modes of barrier failure terminating in eczematous pathology. Intrinsic genetic influences, including mutations that disrupt filaggrin function or structural defects in the stratum corneum are fundamental to barrier disruption in atopic dermatitis (AD). In contrast, for stasis dermatitis, it is often due to a mechanical stretch and hydrostatic pressure from venous insufficiency that leads to barrier disruption [49]. Recurrence of the condition is a major clinical burden, with an incidence ranging from 16% to 53%. Studies indicate that the recurrence is comparatively high in hospital-based settings with respect to that in communities and among hospital patients demanding longer periods of long-term hospitalisation. The predisposing factors for recurrent cellulitis are multifaceted and include demographic variables, environmental factors, and a set of clinical conditions [50]. Due to the concurrent comorbid disease and lymphatic changes, the incidence of cellulitis is accelerating. Risk factors for recurrent cellulitis include dermatomycosis, chronic oedema, venous disease, and obesity [35]. Pathological implications of cellulitis are evident in the lower limbs in about 88% of reported cases [51], with a well-defined set of risk factors, including venous insufficiency, interdigital intertrigo, lymphoedema, obesity, and previous episodes of occurrence. These factors create a unique microbiological and immunological environment that differs from conditions affecting the face, eyes, or upper limbs. Importantly, recent serological studies have shown that *Streptococcus dysgalactiae* subspecies *equisimilis* (SDSE) is the leading cause of lower limb cellulitis in adults, rather than the more commonly known *Streptococcus pyogenes* or *Staphylococcus aureus* [52].

### 4.2. Role of Chronic Edema and Lymphatic Stasis in Cellular Susceptibility

Lymphoedema is one of the most identified risk factors for cellulitis. Secondary lymphoedema normally occurs after radiotherapy or lymphadenectomy, with predisposing conditions such as fibrosis, oedema, and chronic inflammation. Moreover, lymphoedema patients often are imprinted with an impaired immune system, which makes them more susceptible to cellulitis [53]. A cross-sectional study of patients with arm lymphoedema (LIMPRINT) reported a 46% reduction in the risk of cellulitis based on well-controlled lymphoedema, and more advanced stages represent a substantial risk for cellulitis. Additionally, patients with overt arm lymphoedema were at a higher risk of developing cellulitis. These findings could establish the critical role of prevention in progression to lymphoedema to substantially reduce recurrent episodes of cellulitis [54]. Furthermore, a retrospective study in a lymphoedema-specific cohort (2920 patients with breast cancer-related lymphoedema) reported 418 episodes of cellulitis in 231 patients, highlighting the relationship between degree of lymphatic impairment and recurrence risk in the group [55]. After the initial barrier breakdown, both genetic and mechanical disruptions lead to microbial dysbiosis. This condition is a key factor that drives the dermatitis phenotype. In both atopic dermatitis (AD) and stasis dermatitis, the damaged epithelial structure loses its natural chemical defences. This change allows pathobionts, especially *Staphylococcus aureus*, to grow excessively. This microbial imbalance activates pathogen-associated molecular patterns (PAMPs), which interact with pattern recognition receptors (PRRs) on keratinocytes and immune cells. The resulting inflammatory response, driven by toxins and Th2-skewed hypersensitization, creates a cycle of ongoing eczematous inflammation. This shows that whether the initial cause is genetic or mechanical, microbial dysbiosis serves as the common link that drives the clinical signs of dermatitis [49].

### 4.3. Etiological Agents in Cellulitis

Even though cellulitis is a condition that is strictly characterised by erythema, warmth, swelling, and pain, the pathogenesis that is associated with the condition is essentially the result of complex host–pathogen interactions. Recent studies have shown that cellulitis is not, as previously thought, a condition that is essentially caused by recurrent infections in the skin; instead, it is likely that pre-exposure to bacteria prior to the development of the condition can lead to reprogramming of the cells that normally reside within the skin, predisposing the skin to more frequent flare-ups of the cellulitis [56]. Supporting this notion, a study demonstrated that prior inflammatory exposure leads to persistent epigenetic alterations in skin epithelial stem cells, permitting heightened responses to subsequent challenges even when the initial stimulus has resolved [9]. Furthermore, inflammatory memory has been shown to be maintained by stable chromatin remodelling programmes that can be reactivated following subsequent inflammatory challenges, supporting the idea of long-term tissue priming, and there is growing evidence that suggests that these infections can lead to long-term changes to the skin cells and their responsiveness to trained immunity [3,57].

Epidermal colonisation with *Staphylococcus aureus*, clinically presenting as impetigo, is a considerably more frequent training stimulus in atopic dermatitis patients than cellulitis, but the mechanistic basis for focusing on cellulitis as a primary driver of trained immunity remains to be clarified. Impetigo, a disease of the epidermis, triggers mainly keratinocyte-driven innate responses and surface pattern recognition. Cellulitis, a deeper dermal infection, conversely, incorporates a wider array of immune cells in the skin such as dermal macrophages, monocytes, and myeloid progenitors, leading to more widespread and spatially extensive epigenetic reprogramming [58]. Moreover, the dermal inflammatory conditions created during cellulitis facilitate the systemic transmission of trained immunity signals to bone marrow progenitors through circulating cytokines and PAMPs, establishing a more enduring and extensive innate immune memory. This difference does not negate the potential contribution of impetigo to trained immunity induction, but rather emphasizes the fact that cellulitis, with its deeper tissue involvement and systemic immunological footprint, is a mechanistically more impactful training event that can establish the chronic inflammatory continuum characteristic of atopic dermatitis.

### 4.4. Acute Phase—PRR Activation

The predominant etiological agents of cellulitis are β-haemolytic streptococci, specifically the species *S. pyogenes*, and *S. aureus*. The serological analysis of the blood of patients with cellulitis of the lower limbs revealed higher titres of antibodies reacting with streptococcal antigens such as streptolysin O, ScpA and SpyAD [59,60]. Antigenic characterisation of the pathogens has revealed several virulence-associated proteins and toxins present in both *S. aureus* and *S. pyogenes*. The fact that cellulitis often recurs in individual patients indicates that the innate immune system of those patients is often stimulated by the invading pathogen, making it susceptible to immune reprogramming.

Keratinocytes function as active immune sentinels rather than passive barrier cells. Their pattern-recognition receptors allow them to detect bacterial invasion and trigger inflammatory signalling. Toll-like receptor 2 (TLR2) is a major receptor involved in the recognition of Gram-positive bacteria and responds to lipoteichoic acid (LTA), peptidoglycan (PGN), and bacterial lipoproteins. Experimental studies have demonstrated that purified LTA and PGN from *S. aureus* induce high levels of TNF-α production in human monocytes by activating TLR2-mediated NF-κB and MAPK signalling pathways [61]. Although TLR4 is known to specifically recognise Gram-negative bacterial lipopolysaccharides, it may also have an indirect role in the induction of inflammatory responses in Gram-positive infections. Concomitantly, the intracellular recognition of PGN-derived fragments by NOD-like receptors is an additional layer of immune recognition. Muramyl dipeptide activates NOD2, while meso-DAP-containing fragments activate NOD1, both of which activate RIPK2 signalling and the amplification of the inflammatory response [62]. NOD2 signalling plays roles that extend beyond acute pathogen recognition. In the long term, it contributes to immune modulation by regulating the production of defensins and by maintaining cutaneous immune homeostasis through supporting barrier integrity, balanced cytokine production, and proper function of skin-resident immune cells.

C-type lectin receptors on myeloid cells also play a role in pathogen recognition and the regulation of immune responses during skin infection. In addition to receptor activation, sustained exposure to bacterial components can induce persistent production of pro-inflammatory cytokines, including IL-1β, IL-6, and TNF-α, thereby reinforcing inflammatory feedback loops that promote prolonged immune activation. Lipoteichoic acid, a major structural polymer found in Gram-positive bacteria, is released during bacterial growth or lysis and is a potent inflammatory mediator [63]. Peptidoglycan turnover leads to soluble muropeptides that can be released systemically and activate intracellular receptors [64]. Bacterial toxins and secreted enzymes also mediate tissue damage and immune activation. In addition to structural components, bacterial metabolites produced during infection can modulate host cell metabolism and signalling pathways.

### 4.5. Chronic Imprinting

**Figure 4:**
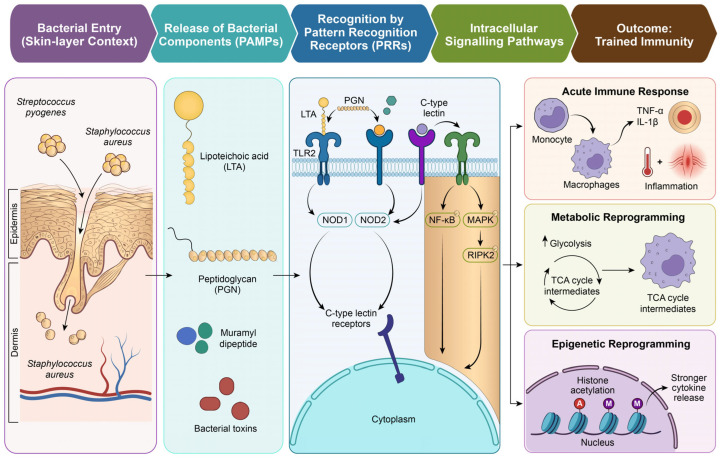
Trained immunity in the development of cellulitis: Pathogen invasion (*Staphylococcus aureus* and *Streptococcus pyogenes*) into the skin releases pathogen-associated molecular patterns (PAMPs) that are detected by pattern recognition receptors (PRRs) including TLR2, NOD1, and NOD2. Engagement of these receptors activates intracellular signalling pathways such as NF-κB and MAPK, initiating acute inflammatory responses. Following this initial phase, innate immune cells undergo metabolic and epigenetic reprogramming. These long-term cellular alterations give rise to a trained immune state, characterised by heightened inflammatory responsiveness upon subsequent stimulation.

Recent studies show that certain microbial signals can shape trained immunity, which is mediated through persistent metabolic and epigenetic changes. Another important feature of trained immunity is the shift of cells towards aerobic glycolysis, which is mediated by the mTOR-HIF-1α pathway. The intermediates of the tricarboxylic acid cycle surge within the immune cells during trained immunity to act as cofactors for the enzymes that control the epigenetic regulation of those cells. In experimental models, after the first exposure to *S. aureus*, they subsequently respond more strongly to a second challenge by displaying higher glycolytic activity, increased histone acetylation, and enhanced cytokine production, all of which point to a functionally “trained” immune state [65]. Recent research into trained immunity in the context of skin infections has shed more light on how bacterial exposure can alter the immune system [66]. When skin barrier integrity is compromised by pathogens like *S. aureus*, acting initially as epidermal commensal organisms, PAMPs of these bacteria interact with immune cells and activate PRRs on immune cells such as TLR2, NOD1/NOD2 and C-type lectin receptors. Activation of these receptors leads to the activation of specific signalling pathways within these immune cells such as the NF-κB, MAPK and RIPK2 signalling pathways, which activate the cells to produce pro-inflammatory cytokines such as TNF-α and IL-1β and thus initiate an inflammatory response to the invading pathogens. Additionally, the immune cells also alter their metabolism and epigenetics in response to the recognised threat from those invading bacteria. Changes in chromatin structure and systemic reprogramming of bone marrow progenitors can lead to prolonged shifts in immune function. Some of the metabolic by-products of the immune system’s response to bacteria such as *S. aureus* contain the intermediates of the tricarboxylic acid cycle, which act as cofactors for the epigenetic enzymes. Thus, the metabolism of bacteria influences the epigenetic regulation of the immune system’s cells. Furthermore, the epidermal stem cells also appear to be able to maintain an inflammatory memory within their cells, allowing them to respond to the same antigen that was initially encountered. Thus, trained immunity explains how these pathobionts can alter the skin immune system. A brief illustration showing the bacterial triggers in cellulitis is shown in Figure 4.

### 4.6. Trained Immunity in Cellulitis: Beyond Preclinical Models

While *S. aureus* is known to be a strong trigger for trained immunity, causing both metabolic and epigenetic shifts in local skin areas and centrally in the bone marrow, a significant knowledge gap exists regarding the precise memory responses in skin-resident immune cells. Apparently, literature that investigates the trained immunity state of innate immune cells in patients with human cellulitis, whether during active disease or between flare-ups, is scarce. Current mechanistic evidence is predominantly based on murine model studies where prior exposure to *S. aureus* primes the innate immune system, which leads to monocyte recruitment, better bacterial clearance, and enhanced wound healing following secondary infection. However, it is worth noting that skin infections also reduce macrophage lifespan, which diminishes memory persistence [41]. This is a crucial limitation, especially considering the weeks-to months intervals in cellulitis recurrence.

However, it is noteworthy that these observations were obtained from isolated peripheral blood monocytes in vitro, rather than from actual patients with cellulitis. Adding to the complexity, the connection between trained immunity and recurrent *S. aureus* infections is not depicted as protective. In fact, activating trained immunity has promoted *S. aureus* persistence in secondary infections, diminishing the effectiveness of antibiotics and subsequently heightening disease severity. This was largely contributed to by the accumulation of fumarate triggered by trained immunity [67]. Moreover, dermal macrophages have a relatively brief lifespan and turn over rapidly during infections, which limits the duration of memory response. For trained immunity to last in barrier tissues, it must be initiated at the hematopoietic stem cell and myeloid precursor levels.

Apparently, another distinction between lower limb cellulitis and other presentations has significant implications for our understanding of trained immunity. Recurrences can be evident in different areas, like the upper limb after a mastectomy with lymph node removal. This suggests that the risk factors and local immune responses vary by location, leading to different recurrence patterns [68]. The importance of distinguishing these factors is highlighted by the high rate of misdiagnosis: both true cellulitis and pseudocellulitis most frequently occur in the lower limb, making up 90.1% and 93.2% of cases, respectively. Misdiagnosis, often mistaken for stasis dermatitis, eczematous dermatitis, or lymphoedema, can be quite variable, with some studies reporting rates as high as 83% [69]. These misdiagnosis rates could seriously complicate any immunological imprints of cellulitis that lack diagnostic accuracy, as non-infectious inflammatory conditions display a very different immune response compared to actual bacterial cellulitis. This diagnostic overlap is very important in chronic inflammatory dermatoses such as stasis dermatitis, which may be similar to cellulitis (pseudocellulitis) clinically, if it occurs in the absence of an active bacterial infection. Chronic inflammatory changes occurring during these conditions influence local immune responses, which may complicate the interpretation of mechanisms of inflammatory memory [70].

## 5. Trained Immunity in Atopic Dermatitis

The conventional perspective on the transition from chronic atopic dermatitis (AD) to acute cellulitis has changed; it is no longer regarded merely as a physical disruption of the stratum corneum. Instead, it is now conceptualized as a fundamental failure of the resolution phase of the innate immune system. This failure is chiefly attributed to hyperresponsive keratinocytes, which play a pivotal role in organising the primed atopic condition. Single-cell transcriptomics substantiates that these keratinocytes maintain enduring H3K27ac marks at inflammatory gene loci, thereby facilitating the continuous production of alarmins in a state of constant accessibility [8].

When keratinocytes are exposed to pathogen-associated molecular patterns (PAMPs) or damage-associated molecular patterns (DAMPs), they release epithelial alarmins such as thymic stromal lymphopoietin (TSLP) and interleukin-33 (IL-33) that trigger and amplify type 2 inflammatory responses in the skin [71,72]. IL-33 signals via the ST2 receptor and has become a key regulator of immune-cell metabolism, impacting macrophage activation and functional polarization [73]. Recent evidence suggests that IL-33-mediated signalling may induce metabolic reprogramming with increased glycolytic activity, a feature of trained immunity [74]. This metabolic adaptation is often associated with epigenetic changes that sustain the enhanced inflammatory response even after the initial stimulus has disappeared [7]. This may result in a persistent hyper-inflammatory state with decreasing efficiency of antimicrobial defence mechanisms. Consequently, the resulting Th2/Th17 immune deviation actively suppresses the production of host-defence peptides, including cathelicidins (LL-37) [75,76].

Long-term epigenetic changes, which can persist even in the presence of clinical remission, are thought to maintain the persistence of this pathological condition. Research suggests that epigenetic memory in the form of histone modifications and changes in chromatin accessibility in resident skin cells and immune populations may persist in clinically resolved or non-lesional skin. In particular, the activating histone mark H3K4me3 has been linked to trained immunity and sustained transcriptional readiness in monocytes, allowing enhanced inflammatory responses to subsequent stimulation. Thus, the immune system may never completely return to its pre-inflammatory state, leaving individuals more susceptible to repeated disease [3]. This molecular signature is additionally indicated by the hypermethylation of the filaggrin (FLG) promoter, which silences the essential structural genes necessary for barrier restoration. Consequently, this compels the existence of an AD patient into a condition where inflammatory genes remain active, even in non-lesional skin. This inherent hyper-vigilance is essentially non-adaptive, promoting an allergic response while concurrently undermining the chemical defences needed to prevent bacterial translocation [77,78].

The presence of *Staphylococcus aureus* as epidermal commensal organisms serves as a critical factor within this microenvironment, which offers continuous, low-grade stimulation that drives the system towards a critical threshold. The pathogen releases extracellular vesicles filled with toxins that infiltrate deeply into the dermis, enhancing the trained immune response through the epigenetic silencing of filaggrin-processing enzymes [79]. The actual progression to cellulitis is subsequently facilitated by a V8 protease-PAR1 axis, which initiates neurogenic itch and mechanical effects [80]. As the bacteria invade the compromised complexes, innate lymphoid cells (ILC2) and Th17 cells, which were prepared to respond to surface allergens, are rendered ineffective against deep-tissue invasion [81]. The IL-33-mediated breakdown of claudins enables swift paracellular entry, where the impaired and misdirected innate cells are unable to mount a localised neutrophilic response. This metabolic exhaustion, coupled with concurrent epigenetic misdirection, leads to the transformation of surface dermatitis into a clinically significant deep-tissue cellulitis. Consequently, future therapeutic approaches must broaden their scope to address temporary immunosuppression and actively prevent the progression to invasive infections such as cellulitis.

## 6. Antibiotics, Resolution Failure and Immune Memory Persistence

Antibiotic therapy is the gold standard of treatment for acute bacterial skin infections. However, although antibiotics are effective in killing pathogenic microorganisms, they do not have a direct effect on the underlying immune dysregulation, barrier dysfunction or persistent inflammatory pathways that result in disease chronicity and recurrence, particularly in diseases such as atopic dermatitis [82]. This suggests that microbial clearance alone may not be enough to reverse the immunological changes and inflammatory memory induced during chronic or recurrent infection. Accumulating evidence also suggests that prolonged epigenetic reprogramming of innate immune cells and antibiotic-associated modifications of the microbiota can induce long-lasting inflammatory responses and disturbances of immune homeostasis following pathogen clearance [30,83].

### 6.1. The Th2-JAK/STAT Axis and Loss of Antimicrobial Effectiveness

The Th2 cytokines (IL-4 and IL-13) stimulate the JAK/STAT pathway and thereby inhibit the production of antimicrobial peptides, leading to persistent release of keratinocyte (KC)-derived TSLP and chronic immune activation [84]. Antimicrobial peptides (AMPs) such as cathelicidins and β-defensins are downregulated in patients with atopic dermatitis, affecting the innate immune defence. Treatment with antibiotics would reduce the acute infection but would not restore the AMPs’ activity or skin barrier integrity, leading to progressive immune dysregulation and recurrent episodes of cellulitis [85]. Human Th2 cytokines, IL-4 and IL-13, inhibit the production of LL-37 and β-defensins, resulting in reduced antimicrobial defence. The activation of the JAK–STAT pathway together with decreased levels of tight junction proteins like claudin-1 results in an additional loss of the epidermal barrier integrity [86].

### 6.2. Antibiotic-Induced Dysbiosis and Barrier Collapse

Antibiotic consumption leads to dysbiosis in patients with atopic dermatitis, contributing to the depletion of commensals in the skin. This microbial imbalance led to a reduction of metabolites, including short-chain fatty acids (SCFAs) and sphingomyelinase, which in turn alters ceramide formation and elevated cutaneous pH. These alkaline shifts activate Protease Activated Receptor (PAR2), evidently worsening the inflammatory cascade [87]. Moreover, the loss of commensal skin flora like *S. epidermidis* facilitates the increased progression of *S. aureus* with the secretion of ample virulence factors, including α-toxins, proteases and superantigens. This impacts hyperactivation of T cells and negatively regulates gap junction proteins like ZO-1 and occludins [88]. In addition to microbial dysbiosis, certain commensal microorganisms could also mediate disease pathogenesis through aberrant host immune recognition. In susceptible individuals with atopic dermatitis, commensals including *Malassezia* spp. can induce both innate and adaptive immune responses leading to hypersensitivity reactions that further promote cutaneous inflammation. These observations suggest that chronic skin inflammation may result not only from microbial imbalance but also from dysregulated immune responses to normally harmless resident microorganisms [89,90].

### 6.3. Epigenetic Imprinting and Resolution Failure

Recurrence of cellulitis leads to epigenetic reprogramming of macrophages through upregulation of H3K4me3 at promoter regions of proinflammatory cytokines like IL-6 and TNF-α. This trained immunity leads to hyperresponsiveness, causing subclinical levels of inflammation, and interferes with the re-establishment of homeostasis after a crisis [91]. SCFAs like butyrate and propionate regulate immune homeostasis via inhibition of histone deacetylases and enhancement of regulatory T cell differentiation. Thus, the reduction increases T-helper type 2 (Th2) cytokine responses (e.g., IL-4, IL-5, and IL-13) and the levels of immunoglobulin E (IgE), worsening atopic dermatitis [92]. IL-31 serves as the major mediator of the itch–scratch cycle associated with atopic dermatitis. Persistent scratching causes mechanical trauma and breakdown of the epidermal barrier, paving the way for bacterial colonization and other associated secondary skin infections, like impetiginized eczema, which may develop into deeper infections under permissive conditions. Antibiotics remove bacterial triggers, but the underlying cytokine-mediated inflammation will persist, worsening the disease condition [93].

### 6.4. Targeted Therapeutics and the Persistence of Tissue-Resident Memory Cells

Therapeutic strategies are increasingly shifting to achieve high-precision modulation rather than broad immunosuppression. Dupilumab inhibits IL-4Rα signalling, providing a lower chance of bacterial skin infections compared to other immunosuppressive agents like cyclosporine. Conversely, JAK inhibitors like upadacitinib and abrocitinib provide robust anti-inflammatory activity through inhibition of JAK–STAT-mediated effects and eventually through inhibition of JAK receptor signalling [94]. Administration of specific biologics such as amlitelimab and nemolizumab offers targeted pathways instead of suppressing the whole immune system. Additionally, a new non-invasive diagnostic method called ‘RNA-based skin tape stripping’ collects skin cells and performs real-time monitoring of inflammatory mediators like TSLP and IL-33 [95]. Tissue-resident memory cells (TRMs) remain in the site of infection, keeping the immune system in a partially active state, resulting in relapse of atopic dermatitis. TRMs produce cytokines like IL-4 and IL-13, which inhibit the expression of barrier proteins, including filaggrin and loricrin. New therapeutic strategies, including JAK inhibitors targeting the JAK-STAT pathway and OX40-targeting antibodies, interfere with activation and survival of these T cells [96].

## 7. Linking Cellulitis and Atopic Dermatitis Through Trained Immunity

The limiting factor associated with antibiotic therapy to restore cutaneous homeostasis, as implicated in the previous sections, is a fundamental shift in the immunological landscape from acute clearance to persistent imprinting. Thus, this concept of trained immunity establishes a mechanistic bridge connecting acute cellulitis and chronic inflammation features of AD [85].

### 7.1. Pathogen-Induced Innate Imprinting

*S. aureus,* initially colonising the epidermis as a commensal organism, is considered a dynamic, active driver of AD rather than being considered a consequence, forming a two-way communication channel between the skin barrier and host immune system. *S. aureus* produces a cluster of virulence factors including alpha haemolysins, Panton–Valentine leukocidins (PVL), enterotoxins (SEA and SEB), and exfoliative toxins (ETA and ETB). This, in turn, facilitates keratinocyte damage in the epidermis and exaggerates inflammatory responses, eventually breaching the dermis to cause cellulitis. Furthermore, metalloproteases like aureolysin and SspA, a V8 protease, degrade structural proteins and AMPs like LL-37, which compromise the skin barrier and act as a reinforcing factor for relapse of AD [97,98].

Exposure to *S. aureus* induces a reprogramming of skin-resident cells, resulting in the trained state followed by the activation of PRRs to produce pro-inflammatory cytokines IL-10 and IL-36, extending the inflammatory repertoire in the infection site [99,100]. This functional change results in a significant metabolic shift characterised by the induction of mTOR signalling and accumulation of fumarate intracellularly. This, in turn, drives an inflammatory phenotype, and these metabolites act as cofactors promoting epigenetic enzymes, favouring histone modification in the promoter region of inflammatory genes [101]. Consequently, macrophages in the system transform into a hyper-responsive state, maintaining NF-kB signalling, creating a chronic environment [102,103].

### 7.2. Molecular Architecture of AD

At the molecular level, atopic dermatitis (AD) exhibits numerous and complex interactions between innate and adaptive immune responses. In the early stages of the disease, the predominant cytokines produced by T-helper cells 2 (Th2) and 22 (Th22) are interleukin (IL)-4, IL-13, and IL-31. These cytokines potentially drive the imbalanced immune responses, the breakdown of the skin barrier and pruritus. Th1 and Th17 are responsible for producing more cytokines, such as IFN-γ and IL-17, in the advanced stage and an increase in body-wide, chronic and disseminated inflammation [104]. The early activation of innate immune mediators like IL-1β and IL-18 is vitally important for triggering inflammation via the NF-κB pathway and the inflammasome pathway, thereby recruiting and activating immune cells to amplify the inflammatory response. Alarmins derived from keratinocytes, e.g., TSLP, IL-25, and IL-33, promote type 2 inflammatory responses and aid communication between epithelial cells and the immune system. In parallel, cytokines from the IL-1 family (particularly IL-1β and IL-18) and IL-12 contribute to the integrative action of innate and adaptive immune responses by regulating processes such as antigen-presenting cell activation, polarization of T-cells and inflammatory pathways [71].

Additionally, various downstream pathways like NFκB, MAPK, and cGAS-STING are also activated by PRR signalling, allowing for enhancement of inflammasome assembly by regulating the expression of proteins such as NLRP3 and Pro-IL-1β. This will result in the production of fully active forms of both IL-1β and IL-18, as well as the induction of pyroptotic cell death through the action of caspase-1 [105]. This process is further reinforced by basophil-driven IL-33-mediated inflammasome activation, highlighting the importance of early innate immune amplification in disease initiation [106]. In addition to basophils, damaged keratinocytes are an important source of IL-33 following epidermal injury, linking barrier disruption to innate immune activation and helping to explain why irritant dermatitis may predispose individuals to the development of both atopic dermatitis and allergic contact dermatitis [107]. Additionally, cytokines cause a breakdown in the epidermal barrier with the aid of Th2 cytokines, including IL-4 and IL-13, which are produced in excess during allergic or hypersensitivity reactions. These Th2 cytokines inhibit the downregulation of filaggrin, loricrin and involucrin and ceramides; this increases the permeability of the skin, which means that the risk of infection with microbes is significantly increased [108]. The inflammasomes get activated, producing further damage and inflammatory signals, and are triggered specifically by microbes such as *S. aureus*, thus firmly linking the pathogen recognition capabilities of the innate immune system to disease pathology [109]. The self-perpetuating cycle formed by the two processes includes impaired barrier function, stimulating immune activation, and entirely compromising barrier integrity.

### 7.3. Cellular Heterogeneity and Transcriptomic Responses

Beyond molecules, cellular mechanisms are also implicated in inflammation. At the single-cell level, increased frequency of memory T cells residing in the tissue, regulatory T cells and CD8 T cell exhaustion are observed in the chronically affected tissue, which points to chronic sustained immune activation, immune exhaustion and a shift in cell types as part of chronic disease development [110]. Additionally, pathways such as OX40–OX40L signalling also provide additional stimulation: T cells sustain their activation, survive long after initial antigens are cleared, and form long-lived memory cells, leading to chronic inflammation linking adaptive immune responses to barrier disruption [111]. In addition to the previous results, transcriptomic analysis has identified key chemokines, such as CXCL1, CXCL2, CXCL3, and CCL20, as well as hub genes, including PI3 (elafin), that regulate the recruitment of immune cells and maintain barrier integrity through the activation of JAK/STAT signalling pathways [112].

### 7.4. Decoding the Bidirectional Relationship Between AD and Cellulitis

When evaluated under a clinical framework, these conditions do not merely coexist; rather, they exist in a bidirectional loop driving cutaneous dysregulation. There is scarce direct longitudinal clinical evidence demonstrating that treated cellulitis conditions could independently elevate the subsequent risk of AD progression. Thus, this association can be better conceptualised as a mechanistic, bidirectional vulnerability loop. Within this framework, the characteristic AD manifestations including Th2-mediated barrier breakdown, filaggrin deficiency, and suppressed antimicrobial peptide activity could eventually activate extensive permissive pathways for *S. aureus* deep-tissue translocation, activating cellulitis. On the contrary, epigenetic priming (H3K4me3 and H3K27ac marks) characterised by extensive gain of function, which is induced in dermal myeloid cells during cellulitis, augments the background tissue alarm tone, reducing the activation efficiency for subsequent eczematous flares [113].

*S. aureus* occupies a mechanistically central position in both directions, making it the most tractable molecular link between cellulitis and AD. *S. aureus* colonises 30% to 100% of individuals with atopic dermatitis (AD), contrasting with healthy individuals. It exacerbates skin inflammation and allergic reactions by modulating both innate and adaptive immune responses through various mechanisms. Superantigen staphylococcal enterotoxin B, released from AD-affected skin, activates lymphocytes and macrophages, while enterotoxins disrupt the skin barrier and elevate type 2 inflammation [12]. This illustrates the AD to cellulitis trajectory. Furthermore, studies could longitudinally establish that persistent *S. aureus* skin colonisation in early life correlates with heightened atopic dermatitis severity and allergic sensitisation. Furthermore, diminished filaggrin expression facilitates subsequent colonisation via a mechanism involving IL-31-mediated filaggrin downregulation, thereby creating a conducive environment for further colonisation [114]. This time-based relation supports bidirectional relationships.

Trained immunity is the most challenging aspect of how cellulitis-induced trained immunity deals with AD’s Th2 pathology. The role of myeloid cells in the pathogenesis of atopic dermatitis (AD) is highlighted, particularly regarding the alternative activation of myeloid cells, such as monocyte-derived macrophages, within the lesional skin of AD. The activation is marked by a rise in CD163+ populations. Moreover, the importance of innate immunity, particularly the function of myeloid cells, has been acknowledged as a crucial yet frequently neglected element in the initiation and progression of the disease. Myeloid cells enhance the local immune response by secreting chemokines and cytokines, presenting antigens, and promoting the recruitment of effector cells [115].

Almost all peripheral innate immune cells, including self-renewing macrophages that reside in tissue and innate lymphoid cells (ILCs), have been shown to possess trained immunity. The deposition of the H3K4me1, H3K4me3, H3K18la, and H3K27ac histone marks in promoter or enhancer regions of inflammatory response genes results in epigenetic reprogramming. This enables permissive chromatin and facilitates expression [91]. Within skin already primed by AD, this permissive chromatin configuration could decrease the threshold not only for antibacterial responses but also for amplification of type 2 alarmins such as TSLP, IL-33, and IL-25—mediators that lie at the intersection of innate immune activation and Th2 polarisation [6].

Lymphatic insufficiency, biologically interesting but clinically ignored, is an integral component of the bidirectional loop. This lymphatic injury is not only a structural problem with direct immunological consequences. Delayed lymphatic drainage increases the time of interaction between microbial stimuli and skin-resident antigen-presenting cells by reducing the clearance of antigens, DAMPs, and immune complexes from the skin. In addition to promoting trained immunity induction, this prolonged antigen exposure sustains the local inflammatory actions, driving Th2 deviation. Conversely, the Th2-mediated barrier breakdown and microbiome dysbiosis of AD create entryways for bacteria to invade, which precipitate cellulitis episodes and close the loop. Moreover, cutaneous dysbiosis may further exacerbate barrier dysfunction and inflammatory responses in AD. In dysbiotic conditions, commensal microorganisms may act as immune triggers that perpetuate disease progression and increase susceptibility to recurrent infections [116]. Furthermore, recurrent cellulitis was associated with a history of lymphoedema and higher BMI [117]. Both factors are also associated with AD severity independently, suggesting that shared predisposing factors may act as structural amplifiers in both directions simultaneously [20].

Both orientations use the same epigenetic language at the molecular level. In the first case, cellulitis via trained immunity predisposes to AD and produces increased inflammatory responses via gain-of-function epigenetic reprogramming in innate myeloid cells. In the second case, where AD immune dysregulation increases cellulitis susceptibility, it affects barrier defence by loss-of-function epigenetic modifications in epithelial cells, such as decreased filaggrin production and inhibited AMP gene transcription through Th2 cytokine-driven histone deacetylation. Despite considerable advancements in the characterisation of trained immunity, recent observations have revealed that there are considerable missing links in interpreting cell-type-specific variations and the molecular interactions between metabolic and epigenetic pathways. In addition to the aforementioned concepts, antibiotic exposure itself may also play a role in microbiome disruption and immune dysregulation, which have been associated with an increased risk of allergic and inflammatory skin disorders. In contrast, the higher risk of cellulitis in patients with atopic dermatitis may be explained not only by barrier dysfunction and microbial dysbiosis but also the use of topical or systemic immunosuppressive treatments including corticosteroids. Therefore, the association between cellulitis and AD is likely to be multifactorial and warrants further validation [118,119].

## 8. Therapeutic Implications: Targeting Trained Immunity in Skin Disease

The transient transition from anti-inflammatory suppression to reprogramming of innate immune memory can be a promising approach in dermatological therapy. This could be achieved by targeting the epigenetic and metabolic checkpoints of trained immunity, restoring cutaneous health, and creating interruptive strategies for the cellulitis–AD inflammatory flare.

### 8.1. Neuroimmune Modulation and Inflammatory Memory

Recent developments demonstrate the importance of neuroimmune modulation in the maintenance of trained immunity in the skin. More precisely, the role of sensory neurons in encoding inflammatory memory through neuropeptides, which include substance P, calcitonin gene-related peptides, and IL-31, which helps in modulating and enhancing immune activation and cytokine production, is evident [120]. Neuroimmune interactions enhance mast cell degranulation and epithelial response, which reinforce the inflammatory response and maintenance of chronic inflammation in the skin. Persistent neuronal stimulation causes sustained transcriptional and epigenetic changes in immune cells and epithelial cells, which result in neurogenic inflammation. This is of significant relevance in the context of atopic dermatitis, which causes bacterial invasion and results in cellulitis [121]. Studies modulating the neuropeptide signalling to inhibit allergic skin inflammation emphasize the importance of neuroimmune regulation in the cutaneous immune response. For example, microneedle arrays were used to deliver neurokinin-1 receptor antagonists to contact sensitizers, resulting in effective reduction of allergic contact dermatitis. This illustrates the role of neuroimmune pathways in innate immunity and skin inflammation. This is particularly important in atopic dermatitis, where chronic inflammation leads to a defective skin barrier, increasing susceptibility to bacterial invasion and the risk of cellulitis [122]. Therapeutically, the neuroimmune pathways could be targeted by IL-31 inhibitors like nemolizumab or modulating neuropeptide pathways, which could also alter the inflammatory memory and restore immune homeostasis [123].

### 8.2. Precision Medicine Through Multi-Omics and Machine Learning

Moreover, single-cell and multi-omics have progressed in the understanding of trained immunity in skin disease. RNA sequencing, ATAC sequencing, and spatial transcriptomics have helped in elucidating the heterogeneity of the immune cells and the epigenetic changes in the environment of trained immunity. The combination of multi-omics has ensured the identification of disease-specific biomarkers and the prediction of therapeutic outcomes [124]. Machine learning also guaranteed the stratification of the patient groups using the unique immune memory signature. Although machine learning-based approaches have displayed promising performance in biomarker discovery and patient stratification, their routine application in clinical management remains limited. These approaches require further validation in the current models prior to widespread adoption. Studies showed that machine learning-driven diagnostic models, which utilise key gene signatures such as IL7R, KRT16, and CCL18, demonstrated high precision in distinguishing atopic dermatitis lesions across diverse transcriptomic datasets [125].

In this context, artificial intelligence (AI)-driven patient stratification has the potential to confer a transformative shift from traditional clinical observation to a molecularly defined precision medicine model for chronic skin conditions. Rather than treating all patients with broad-spectrum anti-inflammatories, machine learning (ML) algorithms could be utilised to classify individuals based on their dominant immune pathways and unique “immune memory signatures”. These models utilise datasets from single-cell RNA sequencing (scRNA-seq) and scATAC-seq. Moreover, analysing transcriptomic fingerprinting, AI shows promise in accurately distinguishing between active lesions and healthy skin. The applied algorithm could stratify patients into distinct molecular “endotypes”, allowing clinicians to perceive beyond surface-level symptoms. Spatial transcriptomics further enhance the data by providing a 2D or 3D map of molecular changes. Ultimately, machine learning models may forecast which patient categories are at higher risk for disease recurrence and predict the success of specific interventions [126].

### 8.3. Epigenetic Modifications with Pharmacological and Postbiotic Interventions

Epigenetic modifications have a primary position in the process of trained immunity, where the inflammatory genes remain in a transcriptionally active state. Modulation of these epigenetic modifications employing histone deacetylase (HDAC) inhibitors, DNA methyltransferase inhibitors, and bromodomain inhibitors has the potential to reinitialise the maladaptive immune memory. The use of natural compounds and postbiotics has shown the ability to modulate the epigenetic pathway, inhibiting the activation of the inflammasome, especially the NLRP3/caspase-1/IL-1β pathway [127]. The attainment of cell-specific epigenetic modulation remains a major challenge due to the widespread effects of these procedures.

Postbiotic interventions represent a particularly compelling epigenetic reset strategy. The SCFAs such as butyrate, propionate, and acetate, generated by bacterial metabolism, are effective pan-HDAC inhibitors [128]. Butyrate acts as a competitive inhibitor at the zinc-dependent active site of Class I and Class II HDACs, blocking the deacetylation of histone H3 K9 and H3 K27 (H3K9ac) sites in the genes coding for inflammatory proteins [129]. By sustaining histone hyperacetylation at Foxp3 and IL-10 promoters, SCFA-mediated HDAC inhibition simultaneously promotes regulatory T cell (Treg) differentiation and suppresses the Th2-skewed cytokine milieu (IL-4, IL-5, and IL-13) that characterizes atopic dermatitis [130]. The activity of propionate further enhances the benefits by stimulating the free fatty acid receptor GPR41/FFAR3, which triggers MAPK signalling to stabilise Tregs and inhibit mast cell degranulation [131]. Importantly, within the scope of trained immunity, butyrate has been shown to eliminate the H3K4me3 epigenetic marks at the promoters of IL-6 and TNF-α genes in response to repeated stimulation by *Staphylococcus aureus*, thus reverting the hyper-responsive macrophage state without reducing antimicrobial vigilance [132]. This ability to reset epigenetics specifically, shutting down the inflammation-associated sites while maintaining or increasing accessibility of barrier-restitution gene sites like FLG and CLDN1, is what makes SCFAs different from HDAC inhibitors and makes them useful in targeting the cellulitis-AD inflammation pathway.

### 8.4. Metabolic Reprogramming and Microbiome-Derived Metabolites as a Therapeutic Target

Agents that inhibit glycolysis, target mTOR signalling, or disrupt the mevalonate pathway (such as statins) can suppress the production of pro-inflammatory cytokines by modulating cellular metabolism and epigenetic regulatory mechanisms [133]. In addition, certain metabolites, including itaconate, exert immunoregulatory effects by influencing epigenetic enzyme activity and inhibiting pro-inflammatory signalling pathways [134]. The skin microbiome has a pivotal and mechanistically complex role in shaping trained immunity. Dysbiosis—marked by an over-representation of *Staphylococcus aureus* alongside a reduction in commensal species such as *Staphylococcus epidermidis*, *Cutibacterium acnes*, and *Roseomonas mucosa*—alters the baseline signals that typically help limit innate immune training [135,136]. Metabolites produced by *S. epidermidis*, particularly short-chain fatty acids like succinate and lactate, promote a tolerogenic, anti-inflammatory state in keratinocytes and dermal macrophages. Loss of these metabolites removes a key regulatory influence, thereby facilitating NLRP3 inflammasome activation and enhancing trained immune responses [137].

Short-chain fatty acids (SCFAs) are essential in linking our microbiome to immune regulation through the inhibition of histone deacetylases (HDACs). Metabolites such as butyrate and propionate, generated by beneficial microbes, obstruct class I and II HDACs. This obstruction results in heightened histone acetylation at critical sites, including Foxp3, IL-10, and TGF-β. Consequently, this mechanism promotes the differentiation of regulatory T cells (Tregs) while simultaneously restraining the proliferation of effector T cells [132]. Beyond just epigenetic modulation, butyrate also communicates with G protein-coupled receptors like GPR109A and GPR43 found on immune cells in the colon and skin. This interaction kicks off cAMP–PKA pathways that lead to the phosphorylation of the NF-κB p65 subunit, which in turn helps to suppress pro-inflammatory cytokines such as IL-6 and TNF [138]. On the other hand, acetate, the most prevalent short-chain fatty acid (SCFA) in our bloodstream, activates the FFAR2/GPR43 receptor on neutrophils and dendritic cells. This process boosts the early clearance of microbes while keeping excessive inflammatory cytokine production in check. What sets acetate apart is its ability to recalibrate innate immune responses instead of just shutting them down completely [139,140].

**Figure 5:**
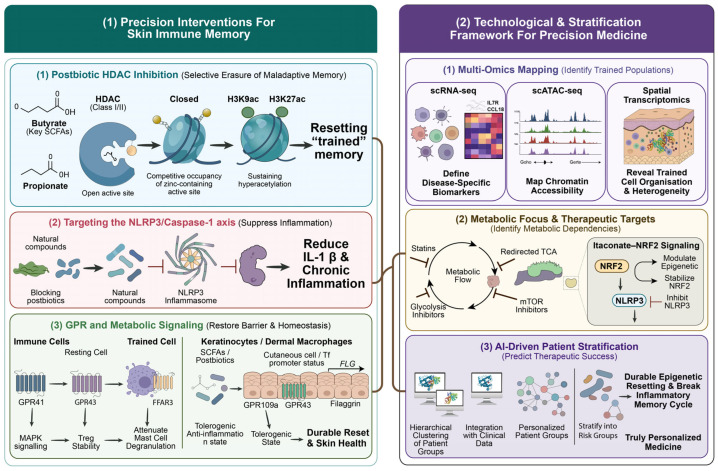
Therapeutic implications for targeting trained immunity in skin diseases. Short-chain fatty acids (SCFAs) like butyrate competitively occupy the HDAC catalytic site, sustaining histone hyperacetylation (H3K9ac and H3K27ac) to selectively erase maladaptive “trained” memory and promote Treg stability. Targeted inhibition of the NLRP3 inflammasome by natural compounds or postbiotics prevents the maturation of pro-IL-1β, effectively silencing chronic inflammatory signalling pathways. Microbiome-derived metabolites activate G-protein-coupled receptors (GPR41, GPR43, and GPR109a) on immune and cutaneous cells to attenuate mast cell degranulation, reduce NF-kB activity, and restore skin barrier markers like filaggrin (FLG). High-dimensional profiling via scRNA-seq, scATAC-seq, and spatial transcriptomics identifies disease-specific biomarkers (e.g., IL7R, and CCL18) and maps the epigenetic “imprints” that allow for hyper-responsive secondary reactions. Interventions targeting the mevalonate pathway, mTOR, or glycolysis restrict the metabolic co-factors necessary for maintaining an open chromatin state in trained cells. Machine learning models integrate multi-omics and clinical data to classify patients into unique “immune memory signatures”, enabling the stratification of individuals into risk groups and the prediction of therapeutic success for durable immune resetting.

In addition to the widely recognized HDAC-inhibiting properties of short-chain fatty acids (SCFAs), the skin microbiome also plays a significant role in trained immunity through several other anti-inflammatory metabolites. These compounds do not merely act in a passive manner; rather, they serve as crucial epigenetic regulators and signalling intermediates. For instance, metabolites such as indole-3-aldehyde and indole-3-propionic acid, which are produced by commensal bacteria that metabolize tryptophan, can activate the aryl hydrocarbon receptor (AhR) on keratinocytes and dendritic cells. Upon activation, AhR signalling facilitates the release of IL-22 while concurrently suppressing essential alarmins such as TSLP and IL-33. This process effectively mitigates the keratinocyte-driven inflammatory cascade that perpetuates trained immunity in AD [141,142]. Simultaneously, metabolites like urolithins and various phenolic acids—derived from the gut–skin microbiome axis—contribute to the reconfiguration of epigenetic patterns. They inhibit the activity of DNA methyltransferase DNMT3A, which may assist in reversing abnormal CpG methylation at the FLG promoter. This partial restoration of filaggrin expression is particularly vital for the repair of the skin barrier, especially following its compromise during conditions such as cellulitis [143]. A brief description of the therapeutic strategies targeting trained immunity in skin diseases is represented in Figure 5.

Taken together, emerging microbiome-based therapies comprising probiotics, postbiotics, live biotherapeutic products, and microbiome transplants converge on a common therapeutic objective: restoring the commensal-derived SCFA and metabolite tone that maintains epigenetic homeostasis and prevents the consolidation of maladaptive trained immunity. Future therapeutic strategies may prioritize enrichment of SCFA-producing microbial communities, alongside the use of HDAC-inhibitory postbiotics, to achieve durable epigenetic reprogramming of skin-resident macrophages and keratinocytes. Such approaches could help interrupt the persistent inflammatory memory that links recurrent bacterial cellulitis to chronic atopic dermatitis. Most evidence currently available supports a role for SCFAs in microbiome-mediated immune regulation, but their widespread anti-inflammatory and epigenetic effects indicate that they could potentially influence inflammatory memory evoked by mild sensitizers common in atopic dermatitis such as propylene glycol, lano-lin, tocopherol and compositae-derived allergens. However, direct experimental evidence to support this effect is still limited and more studies are needed to validate this hypothesis. In this context, trained immunity represents a conceptual shift in dermatology, from short-term symptom control toward long-lasting reconfiguration of immune memory.

## 9. Conclusions and Future Perspectives

The evidence presented in this review suggests a need to modify our classic clinical dichotomy of deep dermal bacterial infections like cellulitis and atopic dermatitis into a spatially stratified continuum with shared epigenetic themes, such as barrier impairment, dysregulated microflora, and trained immune responses. Even though the initiation role for inflammatory responses is confined to the epidermal compartments, deep-tissue bacterial translocation could reiterate a memory response within dermal networks and subsequent bone marrow progenitor cells. This linkage, mediated by *S. aureus*-induced trained immunity between skin barriers, provides a better validated mechanistic model to investigate disease progression from both angles.

Recent advancements in high-resolution technologies have transformed how we comprehend inflammation. For instance, single-cell transcriptomics enables the interpretation of how the inflammatory responses are related to different disease-driving immune cells and different immunological subsets [144]. Meanwhile, ATAC-seq describes long-term immune priming through persistent alterations in chromatin accessibility [145,146], whereas spatial transcriptomics deals with insights into interactions and spatial organisation with immune cells and skin layers [147]. Together, these technologies underline that skin inflammation is not a transient reaction but a persistent, long-lasting cellular and metabolic reprogramming. Crucially, these results suggest how we can redefine inflammation occurring on the skin rather than focusing solely on its histopathology. With this knowledge, it is now possible to classify an inflammatory state depending upon the cytokines produced, modifications in the metabolic pathways, microbiome status, and how these modifications change our level of perception in understanding disease development.

Even though there are recent breakthroughs in identifying the pathophysiological mechanisms, significant gaps remain unaddressed. The definitive relationship between trained immunity and disease development in humans remains intelligible, necessitating a clear demarcation between host defences and maladaptive inflammatory memory. Addressing these challenges demands longitudinal investigations integrating multiomics datasets and extensive clinical records to monitor immune imprinting and spatiotemporal evolution of cutaneous disorders.

These insights support a shift toward precision immune reprogramming instead of broad immunosuppression from a therapeutic point of view [148]. Precision reprogramming offers a therapeutic approach in disease treatment by employing strategies formulated with epigenetic and metabolic pathways alongside microbiome restoration and postbiotic interventions to reverse maladaptive immune memory, all while preserving the patient’s capacity to defend against infections. In addition, incorporating biologics and targeted therapies into this treatment approach may facilitate the delivery of patient-based treatment regimens. Ultimately, a better understanding of how inflammatory memory is established, spatially structured, and maintained functionally in the skin will provide a unifying connection between infection and chronic inflammation.

However, taking this into account, the available literature supports that recurrent bacterial infections, like cellulitis, may contribute to long-lasting changes in the cutaneous immune environment through mechanisms related to trained immunity. Most mechanistic insights into trained immunity are derived from in vitro systems and animal models, with relatively limited validation in human skin disease contexts. To establish how these two methods reinforce each other, their relationship needs to be clarified: For instance, *S. aureus*, which deacetylates filaggrin-regulating histones in keratinocytes (cellulitis to AD), could concurrently acetylate inflammatory gene enhancers in proximal dermal macrophages (AD to cellulitis). Both effects are maintained over multiple infection-resolution cycles and cumulatively reduce the AD flare threshold.

In summary, the linking of the skin microbiome and the response of trained immunity provides a valid model of disease progression between deep dermal bacterial infections and AD. The mechanisms discussed reveal a great need for the research to go beyond symptom-associated treatments and demand a greater shift towards targeted modulation of innate immune memory. By addressing this, it is expected that we can control chronic inflammation and avoid the recurrence of infections, thereby interrupting the progression of the diseases at their root, leading to effective restoration of immune balance.

## Data Availability

No new data were created or analysed in this study. Data sharing is not applicable to this article.

## Abbreviations

The following abbreviations are used in this manuscript:

AAG	AD-associated gene
AD	Atopic dermatitis
AhR	Aryl hydrocarbon receptor
AI	Artificial intelligence
AMP	Antimicrobial peptide
ATAC-seq	Assay for Transposase-Accessible Chromatin with high-throughput sequencing
CCL	Chemokine (C-C motif) ligand
CD	Cluster of differentiation
CXCL	Chemokine (C-X-C motif) ligand
DAMP	Damage-associated molecular pattern
DNA	Deoxyribonucleic acid
ETA/B	Staphylococcal exfoliative toxin A/B
FFAR	Free fatty acid receptor
FLG	Filaggrin
GPCR	G protein-coupled receptor
H3K27ac	Histone H3 lysine 27 acetylation
H3K4me3	Histone H3 lysine 4 trimethylation
HDAC	Histone deacetylase
HIF-1α	Hypoxia-inducible factor 1-alpha
HSPC	Hematopoietic stem and progenitor cell
IFN	Interferon
IL	Interleukin
ILC	Innate lymphoid cell
JAK	Janus kinase
KC	Keratinocyte
LC	Langerhans cell
LPS	Lipopolysaccharide
LTA	Lipoteichoic acid
MAPK	Mitogen-activated protein kinase
MHC	Major histocompatibility complex
ML	Machine learning
mTOR	mammalian Target of Rapamycin
NF-κB	Nuclear factor-kappa B
NLR	NOD-like receptor
PAMP	Pathogen-associated molecular pattern
PAR	Protease-activated receptor
PD-1	Programmed cell death protein 1
PGN	Peptidoglycan
